# Type 2 Immune Mechanisms in Carbon Nanotube-Induced Lung Fibrosis

**DOI:** 10.3389/fimmu.2018.01120

**Published:** 2018-05-22

**Authors:** Jie Dong, Qiang Ma

**Affiliations:** Receptor Biology Laboratory, Toxicology and Molecular Biology Branch, Health Effects Laboratory Division, National Institute for Occupational Safety and Health, Centers for Disease Control and Prevention, Morgantown, WV, United States

**Keywords:** type 2 immune response, lung fibrosis, carbon nanotube, Th2 cell, M2 macrophage, innate immunity, inflammation, cell signaling

## Abstract

T helper (Th) 2-dependent type 2 immune pathways have been recognized as an important driver for the development of fibrosis. Upon stimulation, activated Th2 immune cells and type 2 cytokines interact with inflammatory and tissue repair functions to stimulate an overzealous reparative response to tissue damage, leading to organ fibrosis and destruction. In this connection, type 2 pathways are activated by a variety of insults and pathological conditions to modulate the response. Carbon nanotubes (CNTs) are nanomaterials with a wide range of applications. However, pulmonary exposure to CNTs causes a number of pathologic outcomes in animal lungs, dominated by inflammation and fibrosis. These findings, alongside the rapidly expanding production and commercialization of CNTs and CNT-containing materials in recent years, have raised concerns on the health risk of CNT exposure in humans. The CNT-induced pulmonary fibrotic lesions resemble those of human fibrotic lung diseases, such as idiopathic pulmonary fibrosis and pneumoconiosis, to a certain extent with regard to disease development and pathological features. In fibrotic scenarios, immune cells are activated including varying immune pathways, ranging from innate immune cell activation to autoimmune disease. These events often precede and/or accompany the occurrence of fibrosis. Upon CNT exposure, significant induction and activation of Th2 cells and type 2 cytokines in the lungs are observed. Moreover, type 2 pathways are shown to play important roles in promoting CNT-induced lung fibrosis by producing type 2 pro-fibrotic factors and inducing the reparative phenotypes of macrophages in response to CNTs. In light of the vastly increased demand for nanosafety and the apparent induction and multiple roles of type 2 immune pathways in lung fibrosis, we review the current literature on CNT-induced lung fibrosis, with a focus on the induction and activation of type 2 responses by CNTs and the stimulating function of type 2 signaling on pulmonary fibrosis development. These analyses provide new insights into the mechanistic understanding of CNT-induced lung fibrosis, as well as the potential of using type 2 responses as a monitoring target and therapeutic strategy for human fibrotic lung disease.

## Introduction

Fibrosis is the excess accumulation of the extracellular matrix (ECM), resulting from non-resolving chronic inflammation or exposure to fibrogenic insults (1). The inflammatory and fibrogenic signals elicit an inflammatory wound-healing process that eliminates the damaging agents, mitigates the lesions, and repairs the injured tissue. However, normal tissue repair can evolve into a progressive and irreversible fibrotic process resulting in fibrosis, if the injury is severe or repetitive or if the repair response becomes dysregulated (2). Fibrosis can occur in nearly all tissues and organ systems. Diseases in which fibrosis is a major cause of morbidity and mortality encompass both organ-specific and multisystemic illnesses, with pathologic underpinnings ranging from inflammation and infection to autoimmune dysfunction and foreign body deposition (3). In many cases, fibrosis appears to follow a similar path of progression that resembles wound healing, but is accentuated with or dominated by persistent and exacerbated production and deposition of collagen fibers, leading to the scarring and destruction of organ structures. The critical events and pathways that trigger and govern this irreversible fibrotic progression in organ fibrosis remain largely unclear, which is partly responsible for the lack of effective therapies against fibrotic disease. In this regard, the identification of the important roles of T helper (Th) 2-dependent type 2 immune pathways and the determination of the molecular mechanisms for their activation in fibrosis development in recent years have generated new and significant insights into the pathogenesis of fibrosis, as well as drug targeting for anti-fibrosis therapy (4).

The type 2 immune responses are characterized by the secretion of Th2 cytokines, interleukin (IL)-4, IL-5, IL-9, and IL-13, alongside the activation of Th2 cells, IL-4, and/or IL-13-induced macrophages, and a list of innate immune cells including eosinophils, basophils, mast cells, and innate lymphoid cell (ILC) type 2 cells (5). Because many types of cells are involved in Th2 cell responses, the term “type 2 response” is often used to describe the overall response with Th2 cells playing a central role in the initiation, amplification, and resolution of the response (6, 7). Functionally, type 2 responses were thought to serve as a regulatory mechanism for immunity against extracellular pathogens and for limiting tissue damage by type 1 responses (8, 9). Over the past decade, the scope of type 2 responses has expanded well beyond this type 1–type 2 dichotomy and included a range of functions, such as anti-helminth immunity (10–13), autoimmune suppression (14), neutralizing toxins (6), maintaining metabolic homeostasis (15), and promoting wound repair and tissue regeneration (3, 4), in addition to suppressing type 1 inflammation. Of equal importance, it is now well recognized that dysregulated, hyperreactive, or chronic activation of type 2 pathways can be pathogenic, contributing to the development of a variety of diseases, such as allergic disorders, persistent infection, and tumorigenesis (5). Pertinent to this review, type 2 mechanisms have emerged as an important driver for the development of organ fibrosis, as evidenced in some helminth infections, severe asthma, and many chronic fibroproliferative disorders (3, 16). In this connection, an increasing list of diverse stimuli has been recognized as inducers of type 2 responses to promote fibrogenesis. These include active molecules that damage tissues directly, factors that modulate the balance among Th1, Th2, and Th17 immune functions, and pathogenic or inert agents, such as helminth eggs and silica particles, that have a relatively large body mass and tend to accumulate in tissues persistently.

Carbon nanotubes (CNTs) are nanomaterials made of one-atom thick graphene sheets in the form of tube-like nanostructures, including single-walled and multi-walled carbon nanotubes (SWCNT and MWCNT, respectively). Owing to their excellent mechanical strength, thermal conductivity, and electrical and optical properties, CNTs have been developed with a wide range of applications for both industrial and commercial uses, including electronics, energy production, construction materials, drug delivery, and healthcare products (17, 18). As such, there have been largely increased annual productions of CNTs and CNT-containing products worldwide in the recent decade (19–21). Accordingly, exposure to CNTs is expected to be substantially increased in human populations, including workers that manufacture CNTs and consumers that utilize CNT-containing products (22). The physicochemical features of CNTs, i.e., their nano-scaled size, fiber-like shape, and large surface area, would make them behave as airborne fibers with high respirability. Moreover, CNTs exhibit poor solubility but substantial biopersistence in biological systems. These characteristics are often associated with pathogenic fibers that cause lung and pleural fibrosis and malignancy, as seen in pneumoconiosis and mesothelioma caused by exposure to asbestos fibers (23–26). Indeed, laboratory studies have revealed significant pathologic findings in animal lungs exposed to certain CNTs. These findings raised considerable concerns over the possible health effects of CNTs on humans exposed to CNTs from the workplace, commercial products, and the environment (27–29).

Carbon nanotube-induced pathologic outcomes vary among nanotubes with different properties, but would commonly manifest inflammation and fibrosis, which are similar to those observed in certain human fibrotic lung diseases, such as idiopathic pulmonary fibrosis (IPF) and pneumoconiosis, to some degree. The mechanisms by which CNTs cause inflammatory and fibrotic pathology in the lungs are largely undefined, which greatly hinders the risk assessment of CNT exposure and the development of effective monitoring, prevention, and therapeutic measures against CNT adverse health effects. On the other hand, recent progress has led to the identification of a number of molecular and cellular processes and activities important for CNT lung pathology, some of which have been summarized in several recent reviews (26, 30–36). Notably, CNTs have been shown to elicit significant induction and activation of type 2 pathways in the lungs. Furthermore, increasing evidence supports a critical role of type 2 mechanisms in promoting CNT-induced lung fibrosis. In this article, we discuss recent findings on the induction, activation, and functioning of type 2 pathways during CNT-stimulated lung fibrosis, with a goal to provide new insights into the understanding of the linkage between type 2 mechanisms and fibrosis development in the lungs exposed to CNTs.

## Type 2 Pathways and Lung Fibrosis

The lungs are among the most susceptible organs to fibrosis. As the primary respiratory organ, mammalian lungs perform gas exchange between the blood and inhaled air through a very thin alveolar septal structure that is easily impaired by lesions like interstitial fibrosis and alveolar destruction (37). Moreover, the lungs are constantly exposed to numerous noxious and fibrogenic agents and conditions. These include inhaled microbes, toxic chemicals, and dusts, in addition to bloodborne pathogens and toxicants (26, 38). Some pathogenic agents are preferentially taken up by lung epithelial cells and thereby, accumulate in the lungs to a high level to result in lung-specific lesions from either respiratory or systemic exposure (39, 40). Fibrogenic pulmonary injury also arises from disease conditions, such as cardiovascular malfunction, autoimmunity, idiopathic disorders, and malignancy. On the other hand, the lungs have a considerable functional reserve and a large capacity to repair and regenerate following injury, and thus, are resilient to impairment. However, lung repair can be overwhelmed by the lesions, or become overactive and dysregulated in the presence of persistent or repetitive stimuli, resulting in reduced regeneration but increased fibrosis. These alterations in the lungs would predispose individuals to further infection or injury, forming a vicious cycle to lead to chronic fibrotic conditions. For these reasons, pulmonary diseases with a chronic course and sustained fibrosis are common and are generally very difficult, if not impossible, to cure. Indeed, lung fibrosis represents a major medical concern and a huge burden of healthcare worldwide. Pulmonary diseases in which fibrosis is a major pathologic manifestation encompass a wide range of illnesses with diverse etiology (3, 41). These include allergy (asthma), airway inflammation (chronic obstructive pulmonary disease, bronchiolitis), infection (tuberculosis, pneumonia), pneumoconiosis (silicosis, asbestosis, pleural fibrosis), chemical exposure (bleomycin or paraquat-induced lung fibrosis), genetic disorder (cystic fibrosis), diffuse injury to alveolar cells (acute respiratory distress syndrome), cardiogenic pulmonary lesion (pulmonary hypertension), malignancy (lung cancer, mesothelioma), idiopathic condition (IPF), granulomatous disorder (sarcoidosis), and autoimmune disease (lupus, systemic sclerosis).

The mechanisms by which fibrosis is induced and propagated remain largely undefined. However, a list of triggers and pathways implicated in organ fibrosis has been identified, among which the transforming growth factor-β1 (TGF-β1)- and the type 2 cytokine IL-13-driven pathways are perhaps the most prevailing mechanisms to drive fibrosis development (42, 43). In the above listed lung diseases with fibrosis, immune cells are often activated, with varying degrees of inflammatory and immune response, ranging from innate immune cell activation to autoimmune disease, preceding and/or accompanying the occurrence of fibrosis. It is believed that, under these disease conditions, chronic inflammation brings about an imbalance in the production of cytokines, chemokines, and growth factors, as well as disrupted cellular recruitment in the lungs. Fostered in this pro-fibrotic milieu, an overzealous and persistent immune response of TGF-β1 and/or IL-13 turns a well-controlled reparative mechanism into a pathogenic fibrotic response, leading to lung scarring, alveolar destruction, and ultimately, respiratory failure and death (41).

Transforming growth factor-β1 is one of the first immune factors to be recognized as a key regulator of wound healing and fibrosis. TGF-β1 is a multifunctional cytokine with broad activities, affecting numerous important biological functions, such as embryogenesis, immunity, tumorigenesis, cell proliferation and migration, inflammation, wound healing, and tissue fibrosis (44). With regard to tissue repair and fibrosis, TGF-β1 exhibits both anti-inflammatory and pro-fibrotic activities, including directly inducing the differentiation of fibroblasts into collagen-producing myofibroblasts. In the lungs, TGF-β1 production correlates with fibrosis progression, whereas inhibition of TGF-β1 signaling suppresses the development of lung fibrosis in experimental animal models. In the cases of bacterial and viral infections and bleomycin toxicity, an IL-1- and IL-17A-associated mechanism is activated to propagate the TGF-β1 pathway, forming an IL-1–IL-17–TGF-β1 axis that serves as a major driver of fibrosis during sustained type 1- and Th17-driven inflammatory responses (45).

Type 2 cytokines, especially IL-4 and IL-13, are elevated in many lung diseases with fibrosis, with IL-13 being recognized as having a more prominent role and serving as an attractive drug target for the diseases. IL-13 has been identified as the major driver of tissue repair and fibrosis during sustained type 2 responses induced by allergens, helminths, and fungi, as well as in some lung diseases (3). In these scenarios, activated innate immune cells, such as basophils, eosinophils, mast cells, ILC2s, and macrophages, provide the early secretion of type 2 cytokines, IL-4, IL-5, and IL-13, at the site of initial injury. These cytokines and the local milieu stimulate Th2 cell differentiation, following T cell receptor (TCR) engagement and co-stimulation, and IL-4 receptor α (IL-4Rα) signaling *via* the pathway of signal transducer and activator of transcription (STAT) 6 and GATA-binding protein 3 (GATA-3). Activated Th2 cells then release IL-4 and IL-13 to fuel and orchestrate type 2 tissue repair or, in the presence of persistent injury, fibrosis. IL-13 appears to induce fibrosis both by stimulating the production and activation of TGF-β1 and by directly activating the synthetic and proliferative properties of fibroblasts, myofibroblasts, epithelial cells, and smooth muscle cells (42, 46–48). Like TGF-β1, IL-13 has a dual role in the wound-healing process, as it suppresses inflammation while promoting fibrosis (43). IL-13 can promote fibrosis both by stimulating the production and activation of TGF-β and by directly activating the transformation and function of myofibroblasts (42, 46). Th2 cells producing IL-13 inhibit Th17 responses and IL-13 suppresses Th1 and Th17 inflammation (49). IL-13-driven fibrosis is observed in cases of parasitic egg deposition, fungus and virus-associated pulmonary fibrosis, post-irradiation-induced fibrosis, and silicosis (11, 50, 51). In patients with IPF, elevated production of innate and adaptive immune cell-derived IL-13, as well as IL-13Rα1 and IL-13Rα2, has been detected (52, 53). Moreover, using an animal model of IPF, in which mice with severe combined immunodeficiency were infused with fibroblasts from patients with IPF, a significant reduction in fibrosis and increased repair of the airway epithelium were achieved following an anti-IL-13 antibody treatment; this result supports a critical role of IL-13 in IPF development (54).

Several mechanisms exist to regulate the Th2-driven type 2 responses in tissue repair and fibrosis (5). For example, the type 1-associated cytokine, IL-12, suppresses the differentiation of naïve CD4^+^ T helper (Th0) cells into Th2 cells by promoting the differentiation of Th0 cells toward a Th1 phenotype. Interferon-γ (IFN-γ)-activated M1 macrophages upregulate IL-12 but inhibit IL-10 expression, which also shifts the differentiation of Th0 cells toward a Th1 or Th17 lineage, thus inhibiting Th2 cell responses. TGF-β suppresses both Th1 and Th2-dependent mechanisms, whereas IL-10 strongly inhibits Th1-dependent inflammation (55). In a reciprocal manner, type 2 signals modulate TGF-β and Th1-dependent functions, in which IL-4 and IL-13 stimulate TGF-β production, but suppress Th1-driven type 1 responses. These and other inhibitory mechanisms and cross-regulations are necessary for maintaining the Th2-driven type 2 responses, as well as the TGF-β signaling pathway and the Th1-driven type 1 responses, at levels appropriate for tissue repair. Conversely, a failure or aberrant response in these regulatory mechanisms would lead to an imbalance among the pathways and, consequently, overactivation of Th2 functions to result in fibrosis development (56).

Besides Th2 cells, macrophages are believed to play both regulatory and effector roles in type 2 responses. Macrophages are essential immune effector cells with multiple fundamental functions during inflammation, cell proliferation, tumorigenesis, and tissue repair and remodeling (57–61). Although macrophages have been found to be highly plastic and adaptive both functionally and phenotypically, they are commonly separated into several distinguishable groups or polarization states. Macrophage differentiation is best exemplified by their polarization into M1 (classically activated) and M2 (alternatively activated) phenotypes that are implicated in tissue repair and fibrosis. In this regard, the Th1–Th2 polarization of T cells and the M1–M2 polarization of macrophages have been shown to be tightly controlled and well correlated processes that mirror each other and have a positive feedback between. In type 1 responses, Th1 cells produce a high amount of IFN-γ, which is the major inducing factor to activate M1 macrophages that promote inflammation and confer a killing function. In contrast, in type 2 responses, Th2 cells produce high amounts of IL-4 and IL-13, which serve as the major inducers for the activation of M2 macrophages that suppress inflammation and confer a tissue repair function. Relevant to this review, an overzealous or prolonged M2 polarization and activation of macrophages are associated with fibroproliferative abnormalities, including organ fibrosis and certain cancers (15, 54, 62, 63). Mechanistically, M2 macrophages have been shown to be the dominant effector cells to produce excessive amounts of the major pro-fibrotic mediators, such as TGF-β1, platelet-derived growth factor (PDGF), and tissue inhibitor of metalloproteinase 1 (TIMP1), which promote fibroblast accumulation, fibroblast to myofibroblast differentiation, and ECM production and deposition. Therefore, M2 macrophage polarization and functioning are often considered as a critical effector arm of the Th2-driven type 2 responses in both the initiation and progression of organ fibrosis (64–67).

## CNT-Induced Lung Fibrosis

Carbon nanotube-induced lung fibrosis has been observed and characterized in numerous animal studies over the past decade. As such, CNTs are recognized as a pulmonary fibrogenic agent in laboratory animals, even though different CNTs exhibit variable pathological and toxicological activities due to variations in their physicochemical properties (34, 36). CNTs stimulate a rapid-onset fibrotic response that is detectable as early as day 1 and reaches a peak on day 7 post-exposure. This acute pathology then declines and transits to chronic fibrotic progression afterward. Chronic fibrosis is fully developed on day 28 and prolongs to at least 1 year post-exposure. Therefore, CNT-induced lung fibrosis appears to follow a biphasic course of development toward progressive, persistent, and irreversible fibrosis and destruction of alveolar structures in the lungs.

**Figure 1:**
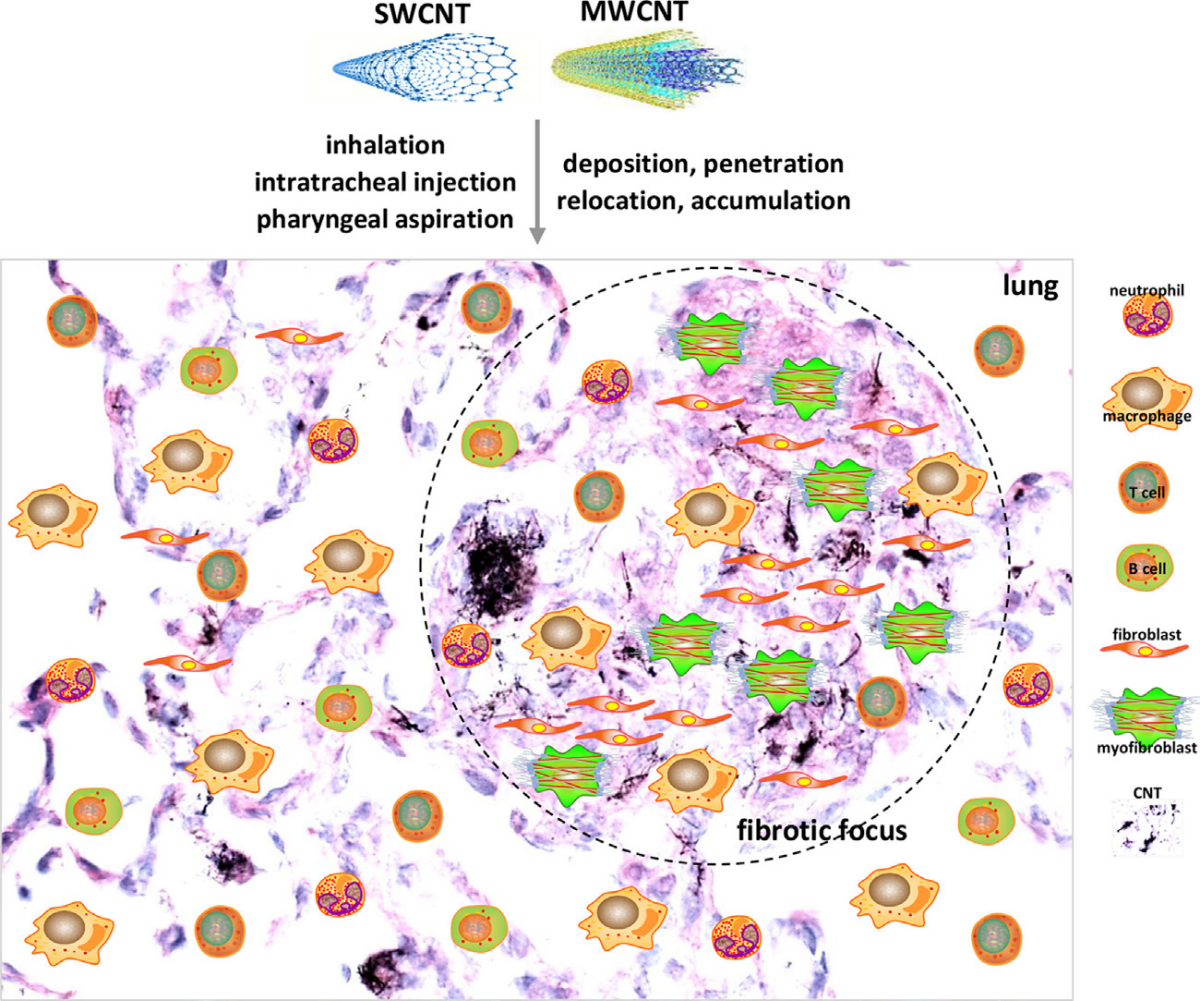
Acute lung inflammation and fibrosis induced by carbon nanotubes (CNTs). CNTs are nano-scaled, respirable fibers with propensity to deposit, penetrate, relocate, and accumulate in lung tissues. Pulmonary exposure to CNTs induces acute-phase responses including a vigorous inflammatory response represented by immune cell recruitment, infiltration, and activation, and a fibrotic response demonstrated by fibroblastic cell accumulation, extracellular matrix deposition, and fibrotic focus formation. Shown are examples of cell types implicated in acute-phase responses as insets on a histopathological image of mouse lungs on day 7 post-exposure to MWCNTs to illustrate acute inflammation and fibrosis. These acute-phase lesions will transit to chronic-phase responses with the occurrence of interstitial fibrosis, epithelioid granuloma formation, and mild inflammation that persist for at least 1 year post-exposure.

The acute-phase response stimulated by CNTs is also highlighted by acute inflammation that starts on day 1 and reaches an apex on day 7 post-exposure (68–70). As hallmarks of acute inflammation, cellularity and infiltration of inflammatory cells, dominated by neutrophils and macrophages, are rapidly elevated in the interstitial, perivascular, and peribronchial regions of CNT-exposed lungs (68–72). T and B lymphocytes are also markedly enriched in the lungs (72, 73). The functions of these immune cells are activated by CNTs, as indicated by the elevated expression and secretion of pro-inflammatory cytokines and chemokines, such as tumor necrosis factor-α (TNF-α), IL-1α, IL-1β, IL-6, and monocyte chemotactic protein-1. Notably, there are also increased productions of pro-fibrotic cytokines, chemokines, and growth factors, such as IL-4, IL-13, chemokine (C-C motif) ligand 11 (CCL11, eotaxin), TGF-β1, and PDGF. These factors likely instruct the rapid deposition of collagen fibers and formation of fibrotic foci around deposited CNTs during the acute-phase response (34, 36, 70, 73) (Figure 1). Therefore, these acute-phase inflammatory responses not only represent the initial pathological reactions to CNT deposition but also cause alveolar and airway injuries and provide the spatial and temporal cues to foster fibrosis development.

During the chronic response to CNT exposure, the interstitial, perivascular, and peribronchial inflammation is reduced to a moderate or mild level, whereas granulomatous inflammation characterized by local accumulation of activated macrophages becomes predominant in the lungs (68, 74). Large amounts of CNT fibers in clusters and collagen fibers in thick bundles are observed in granulomas, rendering granulomas to serve as fibrotic foci in CNT-induced chronic lung fibrosis (34, 75). Interspersed among granulomas is chronic interstitial fibrosis, indicated by increased thickness of alveolar septa with elevated deposition of collagen fibers in the interstitial tissue.

Carbon nanotube-induced lung fibrosis is marked by the presence of mild-to-moderate inflammation, lack of obvious alveolar epithelial cell death, apparent enrichment of fibroblasts and myofibroblasts, thickening of alveolar septa, increased deposition of fibrous ECM, elevated expression of fibrosis marker proteins, and formation of fibrotic foci and epithelioid granulomas. These findings indicate that CNT-induced lung fibrosis resembles the pulmonary response to deposition of fibrogenic foreign bodies, exemplified by insoluble dusts, such as silica and asbestos, and large biologic masses, such as inhaled microbes and invading parasites. At the pathologic level, CNT-induced lung fibrosis demonstrates a high similarity to IPF and to pneumoconiosis (i.e., silicosis and asbestosis), both of which are progressive, incurable, and poorly understood fibrotic lung disease in humans. Recent mechanistic studies have uncovered that the mediators, signaling pathways, and molecular processes involved in CNT-induced lung fibrosis are in agreement with the overall understanding of lung fibrosis in humans and in animal models to a significant extent. These findings suggest a potential of using CNT-induced lung fibrosis as a new animal disease model for studying cellular and molecular mechanisms that may be commonly implicated in fibrotic lung diseases.

## Th2 Activation and Signaling in CNT-Exposed Lungs

A number of recent studies have revealed an association of CNT exposure with increased levels of Th2-type cytokines in the lungs. In the bronchoalveolar lavage (BAL) fluid from mice, the levels of IL-4 and IL-13 proteins were elevated by both SWCNTs and MWCNTs administered *via* intratracheal instillation (71, 76). Inhalation exposure to MWCNTs (XNRI MWNT-7, Mitsui & Company, Tokyo, Japan) induced the mRNA expression of IL-4 and IL-13 in mouse lung tissues (77). An unbiased, genome-wide microarray gene expression analysis of mouse lung tissues revealed that a Th2-type pathway was preferentially enriched by exposure to XNRI MWNT-7 on day 7 after oropharyngeal aspiration, with IL-4 and IL-13 signaling as a predominant phenotype. Time-course studies revealed that IL-4 was significantly induced by MWCNTs at mRNA level on days 1, 3, 7, and 14, and at protein expression on days 3, 7, and 14 post-exposure. IL-13 was significantly induced at both mRNA and protein levels on days 3, 7, and 14 post-exposure, which is slightly delayed compared with the expression of IL-4 (73). The mRNA and protein levels of IL-5, another Th2 cytokine, were also elevated by SWCNTs and MWCNTs in mouse BAL and lung tissues (71, 76–78). Meanwhile, the mRNA and protein levels of IL-25 and IL-33, two type 2 alarmin cytokines produced by damaged epithelial cells to stimulate Th2 cell differentiation and activation, were elevated by MWCNTs in mouse BAL or lung tissues (79–83). A transcriptomic analysis of the gene expression profiles from mouse lungs exposed to three types of MWCNTs supports the notion that a Th2-type response is an integral part of the adverse outcome pathway for MWCNT-induced lung fibrosis (84). Last but not the least, a recent human study reveals significantly increased protein levels of IL-4 in the sputum and serum, and of IL-5 in the sputum, from workers exposed to MWCNTs, compared with controls (28). Taken together, these studies provide substantial evidence demonstrating that Th2-type cytokines are significantly increased in the lungs in response to CNT exposure.

**Figure 2:**
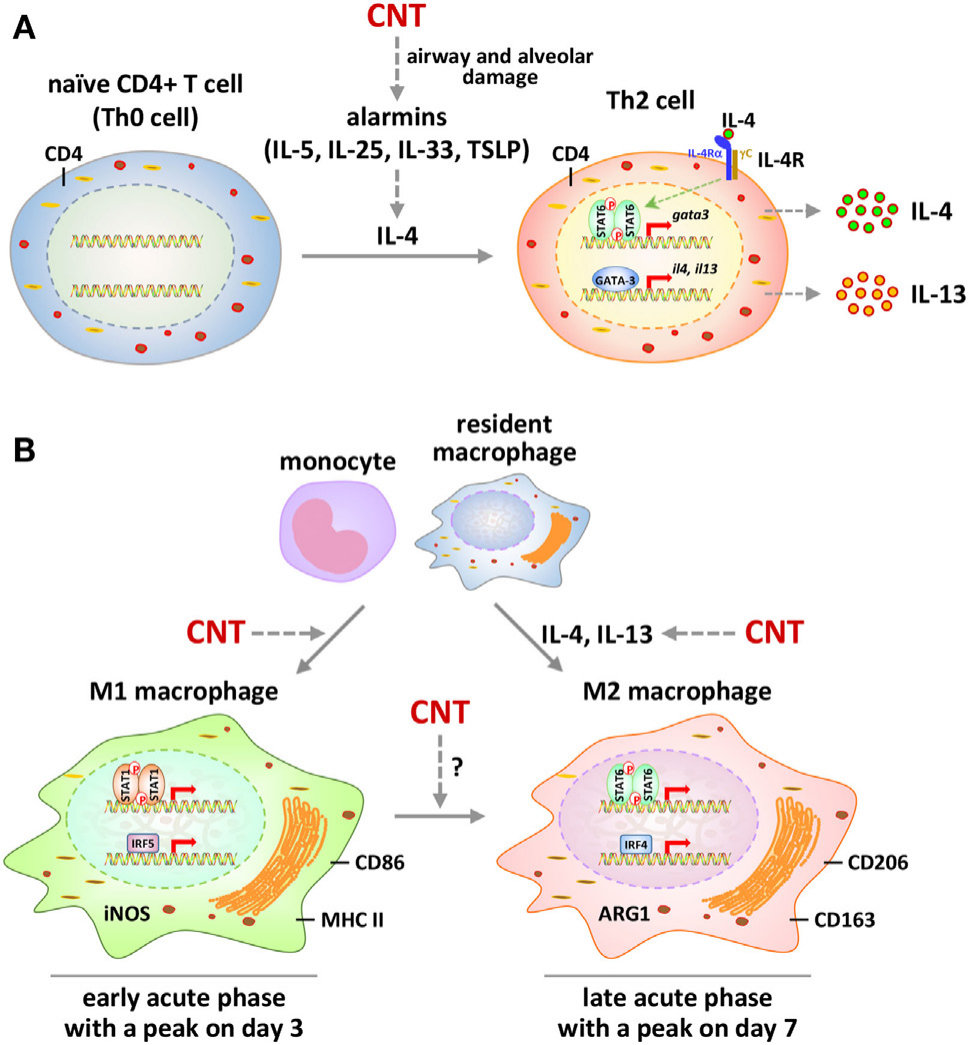
Polarization and activation of Th2 cells and M1-M2 macrophages by carbon nanotubes (CNTs) in mouse lungs. **(A)** Polarization and signaling of Th2 cells. Exposure to CNTs stimulates the secretion of alarmins locally to induce the expression and production of type 2 cytokine IL-4 in the lungs. IL-4 promotes the differentiation of naïve CD4^+^ T cells to CD4^+^ Th2 cells. Activation of Th2 cells by IL-4 is mediated through the IL-4 receptor α/STAT6/GATA-binding protein 3 signaling cascade, leading to amplified production of IL-4 and IL-13. These type 2 cytokines function as the major inducing factors to drive the differentiation and activation of M2 macrophages and myofibroblasts to promote fibrosis. **(B)** Polarization and signaling of M1 and M2 macrophages. CNTs induce the polarization of M1 macrophages from infiltrated monocytes and resident macrophages in the lungs during the early-phase acute response. M1 polarization involves the activation of STAT1 and IRF5 signaling and the production of M1 markers, such as iNOS, CD86, and major histocompatibility complex II (MHC II). During the late phase of the acute response, M1 polarization declines to a lower level, whereas M2 polarization becomes a predominant phenotype. Activation of M2 macrophages is mediated through STAT6 and IRF4 signaling and is marked by the production of M2 markers, such as arginase 1 (ARG1), CD206, and CD163. In addition to the polarization of M2 cells from monocytes and resident macrophages, CNTs may stimulate an M1-to-M2 differentiation of macrophages in the lungs.

Induction of Th2 cell differentiation and activation by CNTs was observed in mouse lungs on days 1, 3, 7, and 14 post-exposure. In these scenarios, exposure to MWCNTs increased the numbers of IL-4^+^CD4^+^ cells and IL-13^+^CD4^+^ cells significantly, indicating the differentiation of Th2 from Th0 cells and the induced expression of IL-4 and IL-13 in these Th2 cells. Notably, MWCNTs activated the IL-4Rα/STAT6 signaling in Th2 cells. Meanwhile, a panel of signature downstream target genes of IL-4/IL-13 signaling, including Il4i1, Chia, and Ccl11, were induced by MWCNTs at both mRNA and protein levels in lung tissues, further supporting the activation of a Th2 response pathway by MWCNTs (73). Induction of Th2 mechanisms by CNTs was also observed in a mouse model of inflammation and fibrosis with Stat6 gene knockout (KO), where the IL-5 level was significantly elevated by MWCNTs (XNRI MWNT-7) in the BAL fluid from wild type, but not Stat6 KO, mice on day 1 post-exposure (78). Together, these findings indicate that, upon CNT exposure, Th2 cell differentiation is induced, Th2-specific signaling pathway is activated, and Th2-type cytokines are increasingly produced, which promote the initiation and progression of lung fibrosis (Figure 2A).

## M1–M2 Polarization and Activation of Macrophages by CNTs

Carbon nanotubes’ nano-scaled diameter and short length, with many in the range of fine (2.5 µm or less) and ultrafine (100 nm or less) particulate matters (PMs), would enable these fibers to be inhaled deep into the alveolar space, to penetrate into the interstitial septa, and even to reach the blood circulation and extrapulmonary organs. Lung resident macrophages, in particular, the alveolar and airway macrophages, are considered to be the first line of the innate immune defense against inhaled particles and fibers that are aspirated into alveoli or deposit on airways. Consistent with this notion, alveolar macrophages with engulfed CNTs, either as separable fibers or in bundles of fibers, are commonly observed in BAL and the alveolar interstitial space of the lungs exposed to CNTs. Macrophages with engulfed CNT fibers are particularly enriched within the acute fibrotic foci and chronic granulomas, indicating a critical role of macrophages in the clearance of CNTs, their transport toward airways and lymphatic vesicles, and their confinement in lung tissues.

The apparent involvement of macrophages in both the acute and chronic responses to CNTs, together with the activation of Th2 pathways in the lungs by CNTs, suggests that CNTs stimulate the formation and functioning of M2 macrophages in the fibrotic response. Direct evidence supporting this notion comes from two separate studies. A recent *in vivo* study examined the M1–M2 polarization of macrophages in the early-phase response to MWCNT exposure in mouse lungs (85). The findings revealed an apparent induction and functionalization of M2 macrophages, occurring on day 3 and peaking on day 7 post-exposure. On the other hand, the polarization and activation of M1 macrophages took place on day 1 and reached a peak on day 3 post-exposure. The M1 polarization was evidenced by the induction of M1 markers, including CD86 (B7-2), MHC II (major histocompatibility complex II), and iNOS (inducible nitric oxide synthase or NOS2), in the lungs and in lung F4/80^+^ macrophages. The M2 polarization was demonstrated by the induction of M2 surface markers, including CD206 (mannose receptor C-type 1 or MRC1) and CD163 (hemoglobin scavenger receptor), as well as functional markers, including arginase 1 (ARG1), FIZZ1 (found in inflammatory zone 1, resistin-like molecule α or RELMα, or resistin-like-α or RETNLα), and YM1 (chitinase 3-like 3 or CHI3L3, or eosinophil chemotactic factor-lymphocyte or ECF-L), in the lungs and in lung F4/80^+^ macrophages. Together, these data reveal the polarization and activation of both M1 and M2 macrophages in mouse lungs by CNTs in a time-dependent manner *in vivo* (Figure 2B).

In a separate study, an oxidized MWCNT preparation was shown to interact with naïve macrophages *in vitro* to induce a mixed M1–M2 phenotype. The M1 phenotype mimicked the response observed in macrophages exposed to lipopolysaccharides, a known M1 inducer, whereas, the M2 phenotype mimicked the response of macrophages treated with a mixture of IL-4, IL-10, and IL-13, three Th2 cytokines known to induce M2 polarization (86). The induction of both M1 and M2 macrophages without a predominant phenotype indicates that CNTs indeed stimulate the M1 and M2 polarization from naïve macrophages directly; however, induction of either M1 or M2 would require additional regulatory signals for it to become a predominant phenotype. In the *in vivo* study discussed above, these regulatory signals were likely provided by the inflammatory milieu in the lungs exposed to CNTs in a time-dependent and sequential manner. These regulatory cues were absent in the *in vitro* system, thus giving rise to a mixed M1–M2 phenotype with cultured macrophages.

Activated M2 macrophages contribute to Th2-dependent immune functions by producing type 2 pro-fibrotic cytokines and growth factors. Some of the M2-produced factors are clearly elevated during the pulmonary fibrotic response to CNTs, including TGF-β1, PDGF, TIMP1, and matrix metalloproteinase (MMP) 12, which indicates the involvement of M2 macrophages in the response to CNTs.

Transforming growth factor-β1 is considered as one of the most predominant endogenous pro-fibrotic factor in organ fibrosis, including CNT-induced lung fibrosis (34). In a number of animal studies, CNTs potently increased the level of TGF-β1 in the lungs. For instance, SWCNTs increased the level of TGF-β1 in BAL on days 7 and 21 post-exposure in mice (68, 87). MWCNTs elevated the level of TGF-β1 in BAL on day 21 post-exposure in mice (88–90). Long MWCNTs (length: 5–15 µm) increased the level of TGF-β1 in BAL on days 7 and 28 post-exposure in mice, whereas short MWCNTs (length: 350–700 nm) did not (91). Also, long MWCNTs (length: 20–50 µm) induced an elevated level of TGF-β1 in BAL on day 1, as well as in alveolar macrophages on day 7, post-exposure in rats (92). The commonly used and well characterized fibrogenic MWCNTs, XNRI MWNT-7, whose length is intermediate with a median length of 3.86 µm, have been shown to significantly increase the level of TGF-β1 in BAL on days 3, 7, and 14, and in lung tissues on days 7 and 28, post-exposure in mice (70, 93). Significantly elevated expression of TGF-β1 in macrophages was also observed on days 7 and 28 post-exposure to XNRI MWNT-7 in mouse lungs, as shown by immunohistochemistry analysis (93). As M2 macrophages are a major source of TGF-β1 during lung fibrosis, the elevated expression of TGF-β1 is likely to reflect the activation of M2 macrophages by CNTs.

Platelet-derived growth factor is another pro-fibrotic growth factor playing an important role in promoting organ fibrosis through multiple mechanisms (34, 94–96). A number of studies showed that PDGF is induced by CNTs in the lungs. MWCNTs significantly increased the level of PDGF-AA, the homodimers of PDGF subunit A, in mouse BAL in a dose-dependent manner on day 21 post-exposure (88, 89). XNRI MWNT-7 significantly elevated the level of PDGF-AA in BAL on days 1, 3, and 7, and in lung tissues on day 7, post-exposure in mice (70). The level of PDGF-AA was induced in BAL on day 1, and in lung tissues on days 1 and 21, post-exposure to MWCNTs in rats (97). These studies consistently report an induction of PDGF in the lungs exposed to CNTs, supporting the polarization and activation of M2 macrophages by CNTs.

Tissue inhibitor of metalloproteinase 1 is highly induced during fibrosis in a number of animal models, such as bleomycin- and paraquat-induced lung fibrosis, and in human fibrotic diseases, such as IPF and liver cirrhosis, implicating TIMP1 and its upregulated expression in fibrosis development (38, 98–101). Previous studies observed increased Timp1 mRNA levels in mouse lungs on day 56, and in rat lungs on days 7 and 30, post-exposure to MWCNTs (92, 102). A recent study showed that XNRI MWNT-7 significantly increased Timp1 mRNA level in mouse lungs during the entire early phase response, i.e., from day 1 to day 14 post-exposure. XNRI MWNT-7 elevated the level of TIMP1 protein in BAL and lung tissues, as well as in macrophages in the lungs, from day 1 to day 14 in mice (103). These data suggest an increased production of TIMP1 by M2 macrophages stimulated by CNTs in the lungs.

MMP12, also known as macrophage metalloelastase 12 or macrophage elastase, is a macrophage-secreted elastase that has been shown to be elevated and confer a promoting function in certain types of fibrosis (104–107). Several studies have shown the induction of MMP12 by CNTs in the lungs. Mmp12 mRNA level was markedly upregulated by SWCNTs in mouse lungs in a time- and dose-dependent manner, as shown by DNA microarray and quantitative reverse transcription-polymerase chain reaction (RT-PCR) analyses. Immunohistochemistry assay detected an increased level of MMP12 protein in SWCNT-exposed lung tissues, with high expression in macrophages (108). Mmp12 mRNA level was markedly upregulated by SWCNTs on days 7, 30, 90, and 180 post-exposure in rat lungs, as determined by DNA microarray and quantitative RT-PCR analyses. Meanwhile, immunohistochemistry demonstrated that the level of MMP12 protein was significantly elevated by SWCNTs in lung tissues on day 90 post-exposure in a dose-dependent manner, with high expression in macrophages (109). Mmp12 mRNA level was significantly upregulated in the BAL cells isolated from mouse lungs exposed to MWCNTs for 60 days (75). Therefore, MMP12 is highly induced in CNT-exposed lungs, especially in macrophages, supporting the activation of M2 macrophages by CNTs in the lungs.

Taken together, these findings corroborate the induction and activation of M2 macrophages by CNTs in animal lungs, providing cellular and molecular insights into the interactions between CNT exposure and M2 polarization to drive fibrosis development in CNT-exposed lungs.

## Mechanistic Aspects of Type 2 Responses in CNT-Induced Lung Fibrosis

### Mechanisms of Th2 Differentiation and Activation Induced by CNTs

The signaling pathways mediating the differentiation and activation of Th2 cells have been characterized, featured by an IL-4Rα/STAT6-mediated signaling in Th2 cells. Upon stimulation, IL-4 binds to Th2 cells through IL-4Rα to induce the phosphorylation of STAT6. Homodimers of phosphorylated STAT6 translocate from the cytoplasm to the nucleus and upregulate the expression of GATA-3. GATA-3 in turn activates the transcription of genes encoding Th2 cytokines, including Il4, Il5, and Il13 (110–112). Accordingly, differentiation of Th2 cells from naïve CD4^+^ T (i.e., Th0) cells, activation of the IL-4Rα/STAT6 signaling pathway, and elevated production of Th2 cytokines in Th2 cells constitute the hallmarks for the onset of Th2-type responses. MWCNTs significantly increased the level of phosphorylated STAT6 and the amount of GATA-3 in the lungs (73). Moreover, increased levels of p-STAT6 and GATA-3 were observed in CD4^+^ cells, with evidently increased numbers of p-STAT6^+^ CD4^+^ cells and GATA-3^+^ CD4^+^ cells in MWCNT-exposed, but not vehicle-exposed, lungs. These findings demonstrate the activation of IL-4Rα/STAT6 signaling in Th2 cells to mediate Th2 differentiation and activation in the lungs by MWCNTs (Figure 2A).

In addition to IL-4R signaling, Th2 differentiation requires TCR engagement and co-stimulation. While dendritic cells (DCs) have been shown to play a central role in Th2-dependent immune activation, the antigen specificity of Th2 cells remains elusive in many Th2-driven type 2 fibrotic responses. Allergens and microbial or parasitic pathogens may induce Th2 cells specific to their antigens during the development of asthmatic, infectious, or fibrotic pathology in the lungs exposed to these pathogenic agents. However, in sterile inflammation where no apparent exogenous pathogen is found, the antigen specificity for Th2 cells is generally not defined. Self-antigens that are exposed following tissue damage may stimulate the activation of Th2 cells locally. Alternatively, Th2 cells may produce IL-13 directly in response to alarmins. In this latter scenario, Th2 cells adopt an innate-like phenotype in response to tissue-derived signals. Several mechanisms have been proposed to explain how the innate immune system senses type 2-inducing stimuli (6). Many pathogens and allergens can be sensed through pattern recognition receptors that recognize pathogen-associated molecular patterns (PAMPs). Proteolytic cleavage of host proteins by a protease activity from a stimulus, or tissue damage and metabolic changes caused by a stimulus, may also be sensed by cells, such as DCs, though the molecular steps that mediate these sensing mechanisms remain unclear. Activated DCs may decode these diverse signals to program Th2 polarization and type 2 immune responses. Additionally, certain subsets of DCs may have an intrinsic capacity to induce type 2 responses after being primed by diverse stimuli. How CNTs stimulate DCs to elicit Th2 polarization remains to be studied, in particular, with regard to antigen specificity and “DC-priming” mechanisms.

### Signaling Pathways Controlling M1–M2 Polarization

The signaling mechanisms mediating M1–M2 polarization have gained certain clarity in recent years (59, 63, 113). Activation of STAT1 and interferon regulatory factor (IRF) 5 promotes the M1 polarization by upregulating the expression of inflammatory mediators that boost pro-inflammatory and cytotoxic M1 functions. On the other hand, activation of STAT6/STAT3 and IRF4 stimulates M2 polarization to confer anti-inflammatory and tissue repair activities of M2 cells. MWCNTs elicited significant induction and activation of STAT1 and IRF5 with a peak on day 3, which correlated with the induction of M1 macrophages, implicating STAT1 and IRF5 signaling in CNT-induced M1 polarization (85). Induction and activation of STAT6/STAT3 and IRF4 by MWCNTs took place on day 3 and peaked on day 7 post-exposure, which correlated with the induction of M2 polarization, thus supporting a role of STAT6/STAT3 and IRF4 signaling in the induction and functioning of M2 macrophages (Figure 2B) (85). Although detailed elucidation of these signaling pathways awaits further investigation, this study identifies major factors and their signaling pathways for CNT-induced M1–M2 polarization in the lungs, providing insights and guidance for future functional and mechanistic studies on M1–M2 polarization elicited by CNTs.

### Alarmins and the Initiation of Type 2 Responses

Alarmins are molecules released from damaged or diseased cells to stimulate immune responses. Various alarmins serve as danger signals to activate distinct subsets of immune cells *via* specific receptors and signaling mechanisms. In the cases of allergen exposure, parasitic, viral, and fungal infections, and wound repair, damaged or stimulated lung epithelial cells of the airways and alveoli, as well as blood vessel endothelial cells, produce and release IL-33, IL-25, and thymic stromal lymphopoietin (TSLP) locally. These alarmin molecules then recruit and activate a set of innate immune cells, including eosinophils, mast cells, basophils, ILC2s, DCs, and M2 macrophages. Among these cells, eosinophils, mast cells, basophils, and ILC2s are believed to provide the initial production of IL-4 and IL-13 that stimulate the differentiation and activation of Th2 cells. Activated Th2 cells in turn initiate, amplify, and propagate type 2 responses by producing and secreting more IL-4 and IL-13.

**Figure 3:**
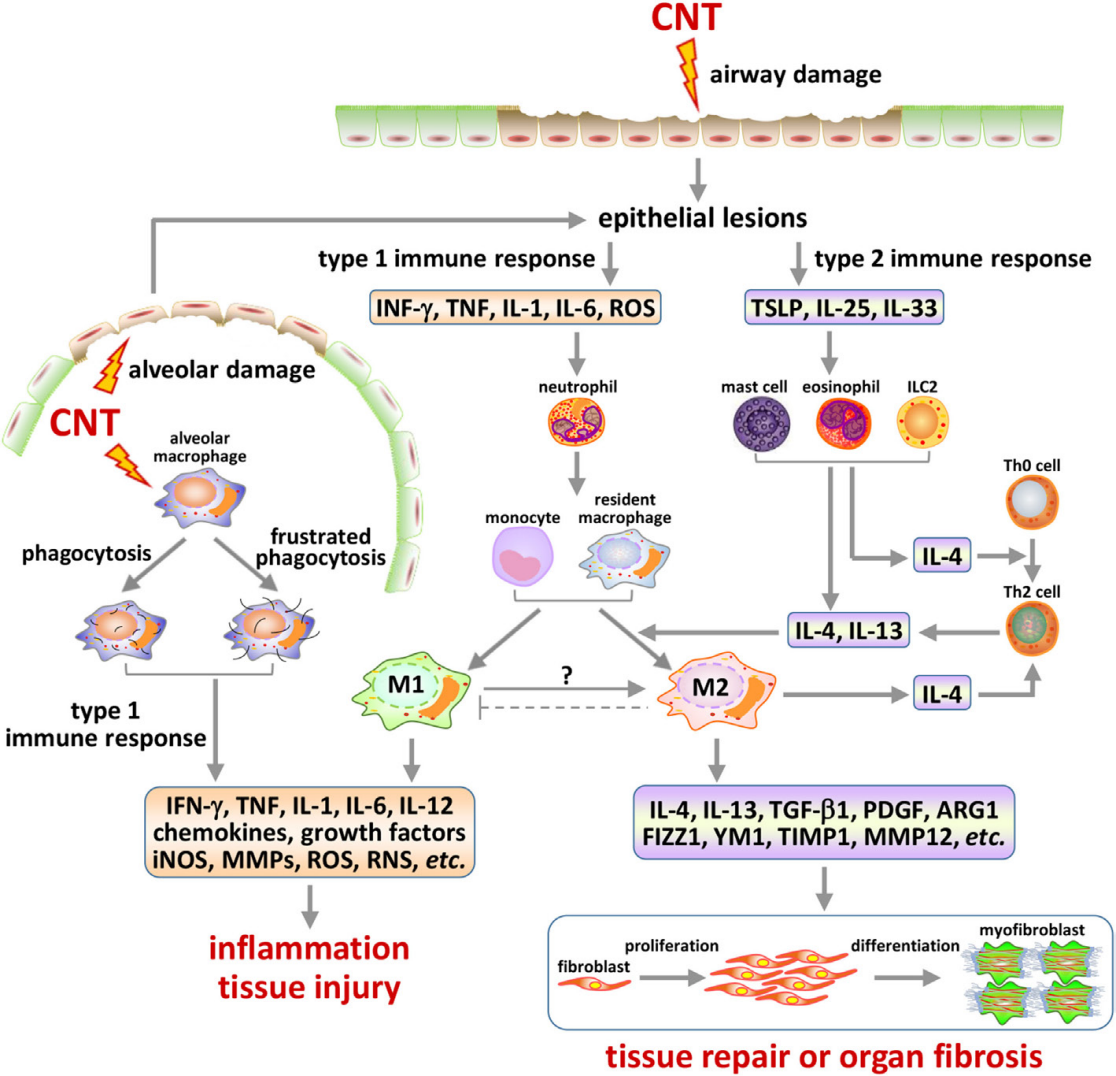
Type 1 and type 2 immune response-associated mechanisms in carbon nanotube (CNT)-induced lung pathology. Inhaled CNTs deposit in airways and in the alveolar space. These nanofibers are capable of inducing epithelial cell lesions *via* multiple mechanisms, such as generating oxidative stress and penetrating cell membrane. The injury of epithelial barriers, such as of the ciliated epithelial and goblet cells in airways and the type II cells in alveoli, results in the production and release of alarmins that trigger either type 1 or type 2 activities in a time and context-dependent manner. CNTs inhaled deep into the alveolar space can be phagocytosed by alveolar macrophages and induce frustrated phagocytosis, both of which serve as another major source of type 1 mediators and alveolar damage. Type 1 responses may consist of the differentiation and activation of Th1 cells and M1 macrophages, which produce pro-inflammatory and cytotoxic factors, resulting in acute inflammation and injury of local tissues. Type 2 responses are marked by the differentiation and activation of Th2 cells and M2 macrophages, which produce anti-inflammatory and pro-fibrotic factors, leading to suppression of acute inflammation and repair of damaged tissues, or organ fibrosis in the presence of persistent or repetitive fibrogenic stimuli. The activation of these immune response-associated pathways, as well as the interplays among them, plays important roles in the initiation, propagation, and chronic development of CNT-induced lung fibrosis.

Emerging evidence reveals that pulmonary exposure to CNTs stimulates the production of alarmins, which correlates with the activation of type 2 pathways in the lungs. IL-33 is constitutively expressed in the epithelial cells of the bronchus and small airways. IL-33 and its receptor (known as ST2, IL-33R, IL1RL1, or IL-1R4) are part of the IL-1 cytokine family, but are unique in that they are associated with the promotion of predominantly Th2, not Th1, responses and are intimately involved in the promotion/maintenance of Th2-associated type 2 inflammation, such as allergy and host defense against helminth infection (114). Oropharyngeal exposure to MWCNTs (50 µg) in mice increased IL-33 in the BAL fluid by ~3.5-fold at 24 h post-exposure (81). In another study, analysis with immunohistochemistry revealed IL-33^+^ type II pneumocytes in the vicinity of alveolar macrophages with phagocytosed MWCNTs or near MWCNT deposits in the alveolar space, but not in alveoli devoid of MWCNTs (81). In separate studies, significantly increased amounts of IL-33 were found in mouse lungs at the mRNA and protein levels, and in the BAL fluid at the protein level, at doses of 1, 2, and 4 mg/kg body weight 30 days post-exposure to MWCNTs (79, 80). In these cases, blocking IL-33 signaling by using anti-ST2/IL-1R4 antibodies or mice with mast cells lacking ST2 significantly reduced type 2 responses, compared with control. MWCNTs also induced IL-33 expression in immortalized C10 mouse lung type II alveolar epithelial cells *in vitro* (81). These findings support a critical role of IL-33 in the initiation of type 2 responses for both airway allergic inflammation and lung fibrosis development (80, 81). In a recent study, an unbiased microarray analysis of the genes induced by MWCNTs in mouse lungs on day 7 post-exposure revealed a significant induction of Tslp gene expression and TSLP signaling, which coincided with the induction of Th2 differentiation, supporting a functional role of TSLP in stimulating Th2 responses (73). In a mouse model of asthma induced by house dust mites, an allergen most frequently associated with asthma in humans, MWCNTs were shown to aggravate the airway inflammation and remodeling, along with some asthmatic phenotypes including eosinophilia and elevated mucus production; these pathologic changes were accompanied by elevated levels of type 2 alarmins, such as IL-25, IL-33, and TSLP, and type 2 cytokines, such as IL-13, CCL12, and TGF-β1 (82). Taken together, available evidence supports that alarmins IL-25, IL-33, and TSLP are induced through lung epithelial cells, in particular, type II alveolar cells, in the lungs by CNTs to foster the development of type 2 responses (Figure 3).

### Type 2 Cells Activated by CNTs

Besides Th2 cells and M2 macrophages discussed above, several other type 2 cells are recruited and activated to function either as regulatory or as effector cells for Th2-driven type 2 responses to CNT exposure in the lungs (Figure 3).

#### Mast Cells

Mast cells play important roles in many innate and adaptive immune responses, in particular, under allergic and some pathogenic conditions. Mast cells sense danger signals *via* several surface receptors, such as PAMP receptors, scavenger receptors, and IL-33 receptor ST2. The role of mast cells and its activation by IL-33 *via* ST2 in CNT-induced lung pathology were investigated using a genetic approach (80). An oropharyngeal exposure of MWCNTs induced (a) lung inflammation, evidenced by increased neutrophils in the BAL fluid; (b) lung fibrosis, shown by increased collagen deposition and granuloma formation; and (c) reduced lung functions, indicated by increased elastic rigidity, reduced compliance, and increased Newtonian resistance in C57BL/6, but not the mast cell-deficient *Kit^W-sh^* or the ST2^−/−^ (ST2 KO), mice. Reconstitution of the *Kit^W-sh^* mice with bone marrow-derived mast cells (BMMCs) from C57BL/6, but not the ST2^−/−^, mice restored the CNT-induced phenotypes similarly to those observed in C57BL/6 mice. At the molecular level, MWCNTs elevated the level of IL-33 in the BAL fluid; moreover, the BAL fluid from MWCNT-exposed mice induced the expression of downstream cytokines, such as IL-5, in cultured mast cells. In both scenarios, induction of IL-33 and IL-5 required the presence of functional mast cells and ST2. Together, these findings demonstrate that MWCNT-induced pulmonary effects, including fibrosis and reduction of lung functions, are dependent on functional mast cells, the induction of IL-33, and the IL-33/ST2 axis. Additionally, whole-body inhalation of MWCNTs (XNRI MWNT-7) for 4 h in a dose range of 6.2–8.2 mg/m^3^ caused an elevated expression of type 2 cytokines, including IL-4 and IL-13, in C57BL/6 mice 24 h post-exposure. However, the induction was absent or largely reduced in *Kit^W-sh^* mice, implicating a critical role of mast cells in the induction of type 2 cytokines and MWCNT-induced allergic airway inflammation (77).

#### Eosinophils

Eosinophils are commonly recruited to combat parasitic and some other infections. Together with mast cells and basophils, eosinophils also control or modulate allergic responses, such as asthma, in the lungs. An oropharyngeal exposure to MWCNTs (FA 21 MWCNTs) resulted in increased numbers of total cells, neutrophils, and eosinophils in BAL from mouse lungs, which were accompanied by elevated levels of IL-33, IL-5, CCL11, and IL-13, and enhanced airway hyperreactivity (AHR) (81). Notably, the increased recruitment of eosinophils, but not of neutrophils, depended on IL-33/ST2 signaling, as pretreatment with anti-ST2 blocking antibodies significantly reduced the eosinophil, but not neutrophil, recruitment. This eosinophil recruitment was compromised in IL33^−/−^, or IL13^−/−^, but not Rag1^−/−^ (KO of the V(D)J recombination activating gene 1 or RAG-1, lacking mature B and T cells), mouse strain, and was correlated with an increased recruitment of ILCs. These findings suggest that MWCNTs stimulate the secretion of IL-33 to recruit and activate a set of ILCs *via* the IL-33/ST2 axis, which in turn produce IL-5 and CCL11 to recruit eosinophils and boost type 2 responses. In a separate study, an inhalation exposure of XNRI MWNT-7 with 4 h per day for four consecutive days at a dose range of 6.2–8.2 mg/m^3^ induced strong pulmonary eosinophilia in mice, accompanied by induction of Il13, Il5, Ccl11, Ccl24, and Ccl17 gene expression, which supports a stimulating effect of MWCNTs on eosinophilia (77).

#### ILC2s

ILC2s are innate helper 2 cells that are stimulated by type 2 alarmins and secret type 2 cytokines, such as IL-4, IL-5, IL-9, and IL-13, to foster the development of type 2 immune responses during helminth infection and allergic lung inflammation. In the example of MWCNT-induced AHR discussed above (81), IL-33-dependent AHR and inflammation, as well as elevated expression of IL-5 and CCL11 and recruitment of eosinophils, were observed. These changes appeared to be independent of T and B cells, but correlated with the recruitment of ILCs that are lineage negative (F4-80, CD3, CD4, CD8, CD11b, CD11c, CD19, Gr-1, TER119, FcεR1), but CD45^+^, Sca-1^+^, IL-7Rα^+^, and ICOS^+^. Although further analyses of these cells are needed in order to classify them as ILC2s, these findings indeed suggest that MWCNTs recruit ILCs to mediate the recruitment of eosinophils and secretion of certain type 2 cytokines during the development of airway AHR and allergic inflammation.

#### DCs and Basophils

While DCs are necessary for type 2 responses, basophils are also needed as they cooperate with DCs to obtain an optimal Th2 response *in vivo*. However, analysis of DCs and basophils in the context of CNT lung pathology has been scarce. By using an *in vitro* human DC culture, it was shown that a CNT scaffold can instruct the DCs to differentiate and to develop a reduced immunogenic profile, as shown by phenotypic, microscopic, and transcriptional analyses (115). In this scenario, CNTs may have interacted with DCs *via* an adhesion-dependent signaling pathway to influence the antigen-presenting properties of these cells, which, in this case, resulted in a decrease in their immunogenicity. This direct modulation of DCs by CNTs may serve as a mechanism to explain the adjuvant effect observed for some CNTs.

#### Epithelial Cells

As discussed above, epithelial cells in the airways and alveoli, such as type II alveolar cells, play important roles in the initiation of type 2 responses to CNT exposure by producing alarmins, exemplified by IL-33 and TSLP (81). These epithelial cells are also effectors contributing to type 2 allergic and fibrotic phenotypes in airways and the lung parenchyma. For instance, MWCNTs (XNRI MWNT-7) induced airway epithelial cell proliferation and mucous cell metaplasia, which correlated with airway fibrosis, interstitial granuloma formation, and an elevated level of TGF-β1 in the BAL fluid (116). These alterations were exacerbated in the absence of STAT1, implicating STAT1 as a negative regulator of type 2 responses to MWCNTs.

#### Myofibroblasts

Myofibroblasts are considered as the ultimate effector cells in both the type 2 and the TGF-β1 pathway-driven lung fibrosis development. Nevertheless, in addition to being responsible for the production of a major portion of the collagen fibers and the contraction of scars, myofibroblasts are known to produce and secrete many regulatory molecules, including cytokines, growth factors, matrix modulating enzymes, and signaling molecules. Therefore, myofibroblasts play, perhaps, more regulatory roles in type 2 fibrosis development than previously thought. MWCNTs have been shown to stimulate the differentiation of fibroblasts into myofibroblasts both *in vitro* and in the lungs *in vivo*. The myofibroblasts so formed directly contribute to the pathologic development in both the acute and chronic phases of fibrosis in mouse lungs exposed to MWCNTs, as summarized in a recent review (34).

### Cross-Regulation Among CNT-Stimulated Immune Mechanisms and Pathways

A number of cytokine-driven immune pathways can be activated by fibrogenic stimuli, including the type 1 (IL-1, IL-6, IFN-γ, and IL-12), Th17-driven (IL-17), TGF-β1-driven (TGF-β1), and type 2 (IL-13) inflammatory pathways, which propel fibrosis development in the lungs in inducer, time, and context-dependent manners (3). The IL-1- and IL-17-associated signaling may propagate the TGF-β1-dependent pathway, forming an IL-1–IL-17–TGF-β1 axis to boost the development of pulmonary fibrosis in sustained type 1 inflammatory responses, whereas, IL-13 acts as the major driver of fibrosis in sustained type 2 inflammation *via* both TGF-β1-dependent and TGF-β1-independent pathways. These two core pathways cross-regulate each other in many cases of fibrosis development.

In addition to activating type 2 and TGF-β1 signaling pathways discussed above, CNTs induce the activation of type 1 and/or IL-17-driven inflammation in mouse lungs. A variety of CNTs, either single-walled or multi-walled, stimulate type 1 inflammation in animal lungs. The type 1 inflammation typically occurs abruptly and peaks on day 1, but diminishes rapidly and to a large extent after day 3 post-exposure to a single dose of CNTs, reflecting the acute course of type 1 inflammation. Type 1 inflammation is characterized by elevated expression and protein levels of pro-inflammatory cytokines, such as TNF-α, IL-1β, and IL-6, in the lungs, the BAL fluid, and the plasma, as well as evidently increased phagocytosis. Pathologically, it is evidenced by the recruitment and tissue infiltration of inflammatory cells, such as neutrophils and monocytes. Moreover, increased production and deposition of fibrotic matrix proteins, including Collagen I and fibronectin, are observed, revealing a rapid-onset fibrotic response as an integral component of the acute phenotype induced by CNTs (70). In response to exposure of CNTs (and possibly, other types of particles and fibers), resident macrophages, including the alveolar, interstitial, and airway macrophages, are activated. Activated macrophages proliferate and migrate toward CNT deposits to phagocytize CNT fibers and transport phagocytized CNTs for lymphatic drainage, mucociliary clearance in the airways, or confinement in the interstitial space. The confinement sites typically consist of activated macrophages, myofibroblasts, bundles of collagen fibers, and accumulated CNT fibers in various forms. These CNT confinement sites would later evolve into fibrotic foci or granulomas, as CNTs persist in the lungs.

A recent study revealed that macrophages enriched during day 1 to day 3 post-exposure to MWCNTs are predominantly M1 macrophages, as evidenced by the elevated expression and co-localization of M1 marker proteins, such as iNOS, CD86, and MHC II, as well as the activation of STAT1 and IRF4 signaling, in M1 macrophages (85). The predominance of M1 macrophages in the early acute-phase response is consistent with their functions to phagocytize and remove or confine CNT fibers as foreign bodies. It is believed that many of the acute-phase pro-inflammatory cytokines, growth factors, and matrix remodeling enzymes are produced by the M1 macrophages. In addition to stimulating type 1 inflammation, these regulatory signals suppress Th2 activation and antagonize type 2 functions. However, in the presence of a persistent stimulus, such as deposits of insoluble CNT fibers, type 1 inflammation may cultivate a microenvironment that would foster Th2 activation, M2 polarization, and type 2 inflammation and fibrosis development, as observed on day 7 post-exposure to CNTs (70). CNT-induced type 1 inflammation and M1 phenotype rapidly diminish after 3 days post-exposure, which correlates with the rise of Th2 and M2 cells, and type 2 inflammatory and fibrotic phenotypes. In this scenario, Th2 and M2 signals may suppress type 1 inflammation and promote the transition from acute inflammation to chronic fibrosis in the presence of persistent CNTs.

In a mouse model of allergen (ovalbumin or OVA)-induced lung airway remodeling, MWCNTs were found to exacerbate OVA-induced lung inflammation and mucous cell metaplasia, as well as airway fibrosis and granuloma formation (117). Notably, MWCNTs, coupled with allergen exposure, induced a mixed Th1/Th2/Th17-driven phenotype, in which the expression of Th1 chemokine CXCL10, Th17 cytokine IL-17, and Th2 cytokines IL-13 and IL-5 was significantly elevated. These changes correlated with the exacerbated lung inflammation and goblet cell formation, but not fibrosis, in cyclooxygenase (COX)-2 KO mice. This study demonstrates that MWCNTs are capable of stimulating Th17-driven inflammation in addition to Th1 and Th2 responses. Moreover, the mixed activation of these three immune pathways is, perhaps, necessary for the maximal inflammation and epithelial cell metaplasia observed in the airways and for fibrosis in the lungs.

In aggregate, MWCNTs induce the activation of multiple components of Th2-driven type 2 responses in the lungs in a time and context-dependent manner, to propel type 2 lung fibrosis development. The Th2-dependent type 2 mechanisms of fibrosis are modulated *via* cross-interactions with other cytokine-driven mechanisms, such as the type 1- and TGF-β1-driven pathways (Figure 3).

## Implications of Type 2 Mechanisms in CNT-Induced Pathologic Effects

Several issues regarding the functional roles of Th2-dependent type 2 mechanisms in CNT-induced pathologic outcomes warrant further discussion.

### Fibrosis

One important issue that remains to be addressed is whether and to what extent Th2-driven type 2 responses contribute causatively to the development of CNT-induced lung fibrosis. Current data support type 2 responses as a key mechanism to drive fibrosis development in CNT-exposed lungs. Nonetheless, genetic and immunological evidence through the gain- or loss-of-function type of experiments, such as gene KO, antibody neutralization, and leukocyte transplantation, would be needed to establish a causative relationship between type 2 pathways and fibrosis development induced by CNTs. In this regard, a few studies have provided some answers. For instance, in one study on type 2 alarmins discussed above, mast cells and the IL-33–ST2 axis were shown to be required for CNT-induced lung fibrotic response, granuloma formation, and reduction of lung functions, as these pathologic outcomes were observed in the wild-type, but not mast cell-deficient or ST2-deficient, mouse strain; moreover, reconstitution of the mast cell-deficient mice with the wild-type, but not ST2^−/−^ BMMCs restored the pathologic phenotypes (80). IL-33 was also shown to be critical for MWCNT-induced airway response in mice, as pretreatment with anti-ST2 blocking antibodies largely diminished the Th2-associated airway inflammation induced by MWCNTs (81).

Signal transducer and activator of transcription proteins are associated with the polarization and activation of Th1/Th2 and M1/M2 cells. In one study, greater airway fibrosis and induction of TGF-β1 by XNRI MWNT-7 were observed in Stat1^−/−^ mice, compared with wild-type mice. This finding is consistent with the notion that STAT1 is a suppressor of type 2-driven fibrosis, possibly by boosting the M1 polarization of macrophages, which inhibits Th2 and M2 formation and function (116). On the other hand, STAT6 signaling is the hallmark pathway for the differentiation and activation of both Th2 cells and M2 macrophages. Activation of STAT6 signaling leads to the phosphorylation and nuclear translocation of STAT6. In Th2 cells, phosphorylated STAT6 upregulates the transcription of GATA-3, which then upregulates the genes encoding Th2 cytokines, including Il4, Il5, and Il13. In M2 macrophages, phosphorylated STAT6 directly upregulates the transcription of the genes encoding certain M2 markers, such as Arg1 and Mmp12. A recent study demonstrated that the level of IL-5 in BAL was significantly induced by XNRI MWNT-7 on day 1 post-exposure in wild-type, but not Stat6^−/−^, mice. Moreover, the Stat6^−/−^ mice displayed significantly reduced lung fibrosis on day 28 post-exposure to MWCNTs, compared with the wild-type (78). These findings on the activation of different STAT-dependent pathways in Th2 cells and M1/M2 macrophages likely reflect a causative correlation between type 2 immune activation and MWCNT-induced lung fibrosis.

Osteopontin (OPN), a type 2 mediator protein secreted by M2 macrophages, plays multiple roles in lung fibrosis, such as stimulating TGF-β1 expression and activation, promoting fibroblast to myofibroblast differentiation, and inducing the expression of ECM proteins (93). By comparing between wild-type and Opn^−/−^ mice, MWCNTs were shown to significantly elevate the level of TGF-β1 protein in lung tissues and in lung macrophages in an OPN-dependent manner. Conversely, loss of OPN attenuated TGF-β1 signaling in the lungs and reduced lung fibrosis (93).

Tissue inhibitor of metalloproteinase 1 is another type 2 pro-fibrotic mediator implicated in CNT-induced lung fibrosis. Timp1^−/−^ mice displayed significantly reduced levels of fibrotic focus formation, ECM deposition, fibroblast proliferation, and fibroblast to myofibroblast differentiation in the lungs during the acute-phase response to XNRI MWNT-7, compared with wild-type (103). This finding indicates a critical role of TIMP1 in the initiation and progression of MWCNT-induced type 2 lung fibrosis. TIMP1 may regulate fibrosis development by controlling the expression of a panel of cell cycle-regulating genes, which requires the activation of the TIMP1/CD63/integrin β1 axis and the extracellular signal-regulated kinase (ERK) signaling, to cause elevated proliferation of fibroblasts in type 2 cytokine-primed lungs (103).

### Inflammation

As discussed, CNTs stimulate both type 1 and type 2 inflammatory responses in a time-dependent manner. The type 1 inflammation occurs immediately following exposure, but would largely resolve after day 3 post-exposure. The type 2 inflammation arises on day 3 and reaches a peak on day 7, followed by reduction during the second week to a much reduced, but more chronic and steady, level. These distinguishable, time-dependent patterns of the two types of inflammation correlate well with their roles in CNT removal and tissue damage by type 1 inflammation on one hand, and tissue repair and transition to chronic fibrosis by type 2 inflammation on the other hand. Type 1 and type 2 inflammatory responses also regulate each other at multiple levels and through multiple mechanisms. Mutually inhibitory activities between them are prominent in many fibrotic conditions, which presumably help maintain the inflammatory responses at levels appropriate to the physiologic or pathologic need of the local environment. Inhibition of type 2 inflammation by type 1 mechanisms in CNT-exposed lungs is evidenced in a study on Stat1^−/−^ mice (116). STAT1 is required for M1 polarization and type 1 inflammation. Loss of STAT1 in mice is associated with elevated fibrosis, granuloma formation, and increased type 2 inflammation, thus supporting an inhibitory role of STAT1-dependent type 1 mechanism in type 2 inflammation development. The rise of type 2 inflammation is associated with the rapid decline of type 1 inflammation in the acute-phase response to CNT exposure in the lungs, suggesting that type 2 immune mechanisms promote the resolution of type 1 inflammation. However, direct evidence supporting the inhibition of type 1 inflammation by type 2 mechanisms is currently lacking. Examination of type 1 inflammation in mice with compromised type 2 functions would be necessary to clarify this possibility in future studies.

### Allergic Airway Responses

Allergic asthma is a chronic inflammatory disorder of the airway characterized by bronchial obstruction, AHR, and increased mucus production. CNTs have been shown to cause or potentiate airway asthma-like responses associated with type 2 immune activation in several investigations. In one study, MWCNT exposure resulted in elevated levels of IL-33, AHR, eosinophil recruitment, and production of Th2-associated cytokines and chemokines in the lungs. These events appeared to depend on IL-13 signaling and the IL-33/ST2 axis, as well as a subset of ILCs (81). In a separate study, a short-term inhalation of XNRI MWNT-7 induced innate immunity-mediated allergy-like airway inflammation in healthy mice, in which marked eosinophilia was accompanied by mucus hypersecretion, AHR, and elevated expression of Th2 type cytokines. Moreover, mast cells and macrophages were found to partially regulate inflammation caused by MWCNTs in the early stage of these responses (77).

MWCNTs potentiated airway allergic and fibrotic alterations in OVA-sensitized asthma mouse models (116–118). In these scenarios, pre-existing allergic inflammation and airway fibrotic remodeling were shown to be mutually promoting, resulting in a chronic airway pathologic condition with both allergic and fibrotic phenotypes. These include AHR, increased airway epithelial cell proliferation and mucous cell metaplasia, eosinophilia, an elevated level of serum IgE, airway inflammation and fibrosis, and parenchymal fibrosis and granuloma formation, accompanied by elevated levels of type 2 cytokines. Some of the allergic phenotypes were exacerbated by MWCNTs in Cox2^−/−^ or Stat1^−/−^ mice, compared with wild-type, suggesting that COX2 and STAT1 act as negative regulators to suppress the stimulating effect of MWCNTs on the allergic responses (116, 117). SWCNTs also exacerbated OVA-induced allergic asthma; moreover, this exacerbation was counteracted by concurrent administration of vitamin E. The mechanism of vitamin E inhibition on SWCNT effects involved elimination of ROS, downregulation of Th2 responses, reduction of Ig production, and relief of allergic asthma symptoms (119).

MWCNTs aggravated the house dust mite-induced airway inflammation, remodeling, and asthmatic phenotypes, i.e., elevated levels of total serum IgG1 and allergen-specific IgG1, eosinophilia, and hyper-production of mucus, in mice (82). These asthmatic and fibrotic airway alterations correlated with elevated levels of type 2 alarmins, such as IL-25, IL-33, and TSLP, and type 2 cytokines, such as IL-13, in a dose-dependent manner, thus supporting a critical role of type 2 mechanisms in the allergic response to MWCNTs in mouse lungs challenged with house dust mite allergens.

### Myocardial Ischemia

Exposure to ultrafine airborne PMs is known to cause adverse cardiovascular events in human populations, some of which are associated with certain preexisting cardiovascular conditions. MWCNTs have been shown to cause an exacerbation of myocardial ischemia/reperfusion (IR) injury in C57BL/6 mice dose-dependently at 1 day post-exposure. This exacerbative effect of MWCNTs on IR injury was also observed in *Kit^W-sh^* mice (mast cell-deficient) reconstituted with wild-type BMMCs, but not in *Kit^W-sh^* mice or *Kit^W-sh^* mice reconstituted with ST2^−/−^ BMMCs (80). The ST2^−/−^ mice demonstrated an augmented IR injury by MWCNTs, compared with vehicle; but the increase in IR injury by MWCNTs was less than those observed in C57BL/6 mice and *Kit^W-sh^* mice reconstituted with wild-type BMMCs. These findings revealed that exacerbation of myocardial IR injury by MWCNTs was mediated through mast cells that were in part activated through the IL-33/ST2 axis, supporting a role of type 2 immune functions in the augmentation of myocardial IR injury by CNTs. Whether and how the cardiac, pulmonary, or blood mast cells are activated by MWCNTs to potentiate the IR injury in an extrapulmonary organ remain to be established.

### Comparison With Th Responses Induced by Silica

Given that CNT-induced lung fibrosis resembles the tissue response to foreign body deposition, it is rational to posit that CNT-stimulated Th2 immune mechanisms would similarly take place in lung fibrosis induced by other fibrogenic particles and fibers. Indeed, cumulative data reveal that pulmonary exposure to silica stimulates a number of immune mechanisms including Th1, Th2, and Th17 responses. Mice received a single intranasal instillation of silica particles exhibited upregulation of IL-13, its receptor subunits IL-13Rα1 and IL-13Rα2, and shared receptor IL-4Rα, development of granulomatous lung inflammation, as well as AHR to methacholine; moreover, these silica-induced type 2 responses were significantly diminished by co-treatment with a mutated form of IL-13 Pseudomonas exotoxin (120). Overexpression of IL-10, a type 2 cytokine and a potent inhibitor of type 1 inflammation, in mouse lungs augments lung fibrosis and Th2 responses induced by silica particles through suppressing type 1 inflammatory responses (121). In a separate study, IL-10-producing regulatory B cells (B10) were shown to be induced by silica instillation. Insufficient B10 clearly inhibited Treg and decreased the level of IL-10, which amplified inflammation and attenuated lung fibrosis by promoting the Th1 immune response (122). Silica also modulates allergic airway responses to allergens such as OVA, which involved Th1, Th2, and Th17 mechanisms (123, 124). In aggregate, silica appears to induce Th1 and Th17 inflammatory responses that are transient and are anti-fibrotic; on the other hand, it stimulates a Th2-dependent type 2 response that is pro-fibrotic and promotes granulomatous fibrosis development. In this regard, the immune responses elicited by silica and the interactions among them are similar to those observed in mouse lungs exposed to CNTs. Whether and how the immune mechanisms induced by CNTs and by silica differ from one another are currently unclear and require further investigation.

## Conclusion and Perspectives

A large body of evidence has been obtained over the past decade to reveal that CNTs generate multiple adverse effects in the lungs of mammals, including inflammation, fibrosis, immunopathology, and tumorigenesis. The possibility for CNTs to cause similar pathologic effects in exposed humans is a serious concern, which demands the careful analysis and comprehensive understanding of the mechanistic basis for CNT pathogenicity. Such information is necessary for conducting mechanism-based risk assessment, safety regulation, and prevention against CNT toxicity. From this prospect, we review the findings on a signaling cascade that links CNT exposure to lung fibrosis, i.e., the Th2-dependent type 2 immune pathways, for discussion of CNTs’ pathogenic effects in the lungs. These results represent a current and promising research area that underlies CNT-induced pulmonary fibrosis and is linked to a number of other adverse effects of CNTs. Highlighted among the results are the identification of Th2, M2, and other type 2 immune cells, the type 2 cytokines, and the alarmins induced by CNTs, and the delineation of cross-interactions between type 2 pathways and type 1, Th17, and TGF-β1-driven immune functions in the context of CNT-induced lung fibrosis. These findings have generated significant insights into the involvement and mechanism of action of type 2 responses in CNT-induced fibrosis development at the cellular and molecular levels. Although studies in these directions are at an early stage in general and many issues regarding CNT-stimulated type 2 responses remain largely unaddressed, these findings provide a timely and mechanistic new starting point for the coming investigations of CNT pathogenic effects.

From a mechanistic point of view, exposure to CNTs would activate a number of mechanisms, in addition to the type 1, type 2, and other immune functions discussed above. These include oxidative stress, various cytotoxic and cell-killing mechanisms, genotoxicity, epigenetic effects, and tumorigenesis. It is conceivable that not only will the type 2 mechanisms cross-interact with other immune functions but also they work with these CNT-induced, non-immune mechanisms, in combinations and in time and context-dependent manners, to initiate and propagate the development of CNT pulmonary, as well as extrapulmonary, effects in mammals. Studies on these interactions between immune and non-immune mechanisms and among different immune pathways may generate new insights into how CNTs interact with the biological systems *in vivo*. Given the complexity of these interactions, investigations across multiple disciplines, such as immunology, toxicology, pathology, genetics, cancer biology, and molecular biology, would be required. At the cellular and molecular levels, CNTs induce and activate many events and signaling pathways, including those involved in type 2 responses discussed above. How these cellular and molecular events modulate and lead to the development of CNT-induced type 2 responses and, ultimately, CNT pathologic effects, such as fibrosis and allergic airway responses, remain to be analyzed in greater details. Presumably, a combination of genetic, immunological, and molecular approaches would be necessary for these mechanistic studies. These analyses are expected to reveal new aspects of the function and mode of action of type 2 pathways in CNT pathogenicity.

The identification of type 2 immune responses and the elucidation of the mechanisms underlying their activation and regulation in CNT-induced fibrosis development have translational potentials. In particular, developing type 2 immune pathway-based biomarkers and therapeutics is a rational approach for the combat against possible health effects in exposed human populations. This direction has raised considerable interests for laboratory research, risk assessment and field studies, policy-making, and drug development. In this regard, several putative drugs targeting type 2 inflammation have been developed and are being tested for type 2 inflammation-associated diseases (3, 5). However, these drug candidates have not been tested for CNT-induced effects yet.

The involvement of type 2 immune mechanisms in disease development is rapidly expanding to include a spectrum of human diseases and animal models, ranging from allergic response, autoimmune dysfunction, and parasitic infection to wound healing, tissue fibrosis, and tumor promotion and metastasis. The pathologic features and the molecular mechanisms of CNT-induced lung fibrosis are in agreement with the overall understanding of lung fibrosis derived from human fibrotic lung diseases and animal models to a considerable extent. Therefore, it is expected that studies on CNT-induced, Th cell-driven responses and fibrosis development would facilitate the mechanistic understanding of, as well as the development of new biomarkers and therapeutic agents against, lung fibrotic diseases that include pneumoconiosis, asthma, and IPF, in addition to CNT-induced lung fibrosis.

## Author Contributions

JD prepared the draft manuscript. QM revised and finalized the article. Both authors read and approved the final manuscript.

## Disclaimer

The findings and conclusions in this report are those of the authors and do not necessarily represent the official position of the National Institute for Occupational Safety and Health, Centers for Disease Control and Prevention.

## Conflict of Interest Statement

The authors declare that the research was conducted in the absence of any commercial or financial relationships that could be construed as a potential conflict of interest.

## Abbreviations

AHR, airway hyperreactivity; ARG1, arginase 1; BAL, bronchoalveolar lavage; BMMC, bone marrow-derived mast cell; CCL11, chemokine (C-C motif) ligand 11 or eotaxin; CD163, hemoglobin scavenger receptor; CD206, mannose receptor C-type 1 or MRC1; CNT, carbon nanotube; COX, cyclooxygenase; DC, dendritic cell; ECM, extracellular matrix; ERK, extracellular signal-regulated kinase; FIZZ1, found in inflammatory zone 1, resistin-like molecule α or RELMα, or resistin-like-α or RETNLα; GATA-3, GATA-binding protein 3; IFN-γ, interferon-γ; IL, interleukin; IL-4Rα, IL-4 receptor α; ILC, innate lymphoid cell; iNOS, inducible nitric oxide synthase or NOS2; IPF, idiopathic pulmonary fibrosis; IR, ischemia/reperfusion; IRF, interferon regulatory factor; KO, knockout; M1, classically activated macrophages; M2, alternatively activated macrophages; MHC II, major histocompatibility complex II; MMP, matrix metalloproteinase; MWCNT, multi-walled carbon nanotube; OPN, osteopontin, or secreted phosphoprotein 1 or Spp1; OVA, ovalbumin; PAMP, pathogen-associated molecular pattern; PDGF, platelet-derived growth factor; PM, particulate matter; Rag1, recombination activating gene 1; RT-PCR, reverse transcription-polymerase chain reaction; ST2, receptor for IL-33 or IL-33R, IL-1 receptor-like 1 or IL1RL1, or IL-1 receptor 4 or IL-1R4; STAT, signal transducer and activator of transcription; SWCNT, single-walled carbon nanotube; TCR, T cell receptor; TGF-β1, transforming growth factor-β1; Th, T helper; TIMP1, tissue inhibitor of metalloproteinase 1; TNF-α, tumor necrosis factor-α; TSLP, thymic stromal lymphopoietin; YM1, chitinase 3-like 3 or CHI3L3, or eosinophil chemotactic factor-lymphocyte or ECF-L.
